# Cancer of the Stomach

**Published:** 1905-12-09

**Authors:** 


### CANCER OF THE STOMACH.

In a lecture on this subject, Dr. Fawcett begins by grouping the cases of the disease according to the locality of the lesion. This classification gives rise to four groups.

1. The pyloric region. This is the most commonly attacked. Different authorities credit the pylorus with a percentage of all cases ranging from sixty-one to seventy.

2. The body of the stomach. Next in frequency of involvement comes the body of the stomach, more especially the region in the neighbourhood of the lesser curvature.

3. The cardiac end of the stomach is seldom attacked.

4. The whole stomach. Occasionally the stomach is attacked by a diffuse new growth which leads to the extreme degree of contraction to which the name of " india-rubber bottle stomach " has been given.

The disease, when it affects the pyloric and cardiac ends of the stomach, gives rise to symptoms more or less defined, but when the body of the organ is attacked the evidence is often very vague. When the cardiac end is affected the œsophagus is involved as a rule; as a result, difficulty in swallowing may be a prominent feature. Fagge used to teach that growths involving the cardia originated in the œsophagus, but this is incorrect. Perry and Shaw have demonstrated that all carcinomata of the œsophagus are squamous-celled, while gastric cancers involving the œsophagus are always glandular.

Touching the relation between simple and malignant ulceration of the stomach, the author states that there is no antagonism between the two: Perry and Shaw found that nine out of one hundred cases investigated by them suggested that the malignant ulcer had been grafted on a previously-existing benign one. On the other hand, simple ulcers are most frequent in women, malignant ulcers in men; simple ulcers most often involve the region of the lesser curvature, malignant ulcers the region of the pylorus. The relation, therefore, if there be any, is vague. The years of maximum incidence of the disease are, for males, from the fortieth to the seventieth year; for females, from the thirtieth to the sixtieth. Before the age of twenty gastric cancer is very uncommon; but a considerable number of cases occur in the third decade—according to Osler 4 per cent. of all cases, but according to Perry and Shaw 2.5 per cent. Osler and McCrae have drawn attention to the frequency with which carcinoma of the stomach in young subjects is heralded by an acute onset. The history, in three of their cases occurring in the third decade, describes an onset with sudden pain after food, with vomiting after food, and with pain ensuing after the lifting of a heavy weight, respectively. The second point insisted upon by these authors is the rapidity with which in such young subjects the disease marches to a fatal termination. In Perry and Shaw's series the duration in the case of subjects under thirty varied from five weeks to five months, while the average duration for cases at all ages was eight and three quarter months.

Though the onset is occasionally acute, it is more often insidious and consists of dyspeptic symptoms. " The important fact to remember is that any person past middle age, and especially at about the age of forty-five to fifty, who develops symptoms of dyspepsia for the *first* time without adequate cause should be looked upon with suspicion." Pain is the most frequent symptom, and next to it vomiting and hæmatemesis. Appetite may persist to a surprising degree, though the desire for food may not be gratified owing to apprehension of the pain that follows its ingestion.

In 90 per cent. of cases of gastric carcinoma, free hydrochloric acid is absent from the stomach, and this absence is a valuable diagnostic sign. The cause of this absence was investigated by Moore and others. The conclusion at which they arrived was that it did not depend upon the local action of the lesion upon the stomach cells, since free hydrochloric acid was either reduced in amount or entirely absent in subjects affected by malignant growths whatever the situation of the disease. The test for free acid should be performed about an hour after the ingestion of a meal of carbohydrate and fat, such as tea with milk and sugar and a round of toast and butter. Much proteid in the diet will interfere with the test, for proteids combine with the acid.

The presence of a palpable tumour is reported in a percentage of cases ranging from about 45 to 75. When the new growth produces a palpable tumour this, when situated at the pyloric end of the stomach, is often freely movable in early stages of the disease. As time goes on, adhesions between the affected area and the neighbouring tissues are very likely to obscure the diagnosis. Thus, if the tumour be pyloric in situation and become adherent to the liver, it is not unlikely to be mistaken for a lesion affecting the gall-bladder. Dr. Fawcett urges that caution be employed in prejudging the possibility of removing any given tumour on the ground of its mobility and small size, for both these attributes are liable to be over-estimated by palpation through the abdominal wall. Conversely a fixed and palpable mass does not necessarily connote an advanced example of malignant disease, but may depend upon induration and matting around an old and cicatrised simple ulcer. Thus, the case is quoted of a woman aged forty-four who had suffered from pain after food, associated with vomiting, for a period of three months. A tumour two inches in diameter was to be felt above and to the right of the umbilicus which, when explored surgically, proved to be the legacy of a simple ulcer.

Cancer of the stomach may be present and extensive without giving rise to any local symptoms. Perry and Shaw have collected a number of cases illustrating the accidental discovery of cancer of the stomach at autopsies upon patients dying of quite unconnected diseases. One of these, illustrated by a specimen in the museum of Guy's Hospital, is very remarkable. The specimen in question was derived from a patient who died as the result of a strangulated femoral hernia. There was no history of any antecedent symptoms pointing to disease of the stomach, yet the autopsy showed a large ulcerated growth stretching from just within the pylorus for a distance of five inches along the lesser curvature of the stomach. Both walls of the stomach were involved, and the growth extended to and protruded the serous coat. Such a case as this is of importance, for it helps one to discount the histories of recent illness not infrequently tendered by patients who have obviously been long unwell. When a patient who is markedly cachectic, and presents, perhaps, signs of secondary deposits in the liver, nevertheless assures the physician that he has only been ill a week or two, the inconsistency is very likely to depend upon such a " latent cancer "of the stomach.

Cancer of the stomach may even cause death without being recognised. Such cases are divided by Dr. Fawcett into two clinical groups:—

1. Those in which cachectic symptoms predominate.

2. Those in which the symptoms are due to secondary deposits. In the first group the diseases with which gastric cancer are most likely to be confused are chronic renal disease, pernicious anæmia, and infective endocarditis. The case is mentioned of a man aged fifty who complained of a weakness of some four months' duration accompanied by swelling of the feet and belly. He was much emaciated, and presented signs of effusion in both pleuræ, with ascites, and dropsy of the legs. He died two months later and was found to have a diffuse carcinoma, by which the stomach was much contracted. In the second group the symptoms presented by the occurrence of secondary deposits may lead to such disguises as paralyses due to deposits in the brain or spinal cord, or ascites from peritoneal deposits, to enlargement of the liver or jaundice from involvement of that organ or its ducts.

Enlargement of lymphatic glands is a considerable corroboration of a suspicion of gastric cancer. The glands most frequently enlarged in cases of cancer of the stomach are those above the left clavicle, behind the left sterno-mastoid muscle, and in the left axilla. If the lymphatic glands in these situations increase in size under observation, and the case is otherwise suspicious, the presence of a cancer of the stomach is a likely explanation of the condition.

